# Patellofemoral pain over time: Protocol for a prospective, longitudinal study investigating physical and non-physical features

**DOI:** 10.3389/fspor.2022.1081943

**Published:** 2023-01-11

**Authors:** Ana Flavia Balotari Botta, Marina Cabral Waiteman, Matheus Henrique Maiolini Ducatti, Carmen Lúcia Gomes Garcia, Lucca André Liporoni Bego Farinelli, David Matthew Bazett-Jones, Ronaldo Valdir Briani, Fábio Mícolis de Azevedo

**Affiliations:** ^1^ Laboratory of Biomechanics and Motor Control (LABCOM), Sao Paulo State University (UNESP), School of Science and Technology, Physical Therapy Department, Sao paulo, Brazil; ^2^ University of Toledo, Department of Exercise and Rehabilitation Sciences, Toledo, Ohio, United States

**Keywords:** knee cap pain, movement analysis, pain sensitization, fear of movement, catastrophism

## Abstract

**Background:**

This is a protocol for a prospective longitudinal study that aims to investigate: (1) group-by-time changes over a minimum of 15 months follow-up in patellofemoral pain (PFP) symptoms, biomechanical, muscle function, pain processing, and psychological features; (2) the extent to which changes in biomechanical, muscle function, pain processing, and psychological features are associated with changes in self-reported pain, physical performance measures, self-reported function, health-related quality of life (HRQOL), and physical activity level.

**Methods:**

Individuals with PFP (*n* = 144) and control individuals (*n* = 85) without PFP were assessed at baseline. Outcomes assessed included: 3D kinematics and kinetics during single leg squat, step-down and single leg hop; maximal torque and rate of torque development of hip abductors and knee extensors/flexors; force steadiness of hip abductors and knee extensors; anterior and lateral trunk endurance; pressure pain thresholds at the center of patella and contralateral shoulder; kinesiophobia (Tampa Scale for Kinesiophobia); pain catastrophizing (Pain Catastrophizing Scale); worst self-reported pain (Visual Analogue Scale); physical performance measures (Single Leg Hop Test and Forward Step-Down Test); self-reported function (Anterior Knee Pain Scale); HRQOL (Medical Outcome Short-Form 36), and physical activity level (Baecke’s Questionnaire). Follow-up assessments will be identical to the baseline and will be performed after a minimum of 15 months. Generalized linear mixed model (GLMM) will be used to investigate group-by-time differences. Linear regression models will be used to determine the extent to which changes in biomechanical, muscle function, pain processing, and psychological features are associated with changes in self-reported pain, physical performance measures, self-reported function, HRQOL, and physical activity level.

**Discussion:**

Physical and non-physical features have been previously associated with PFP. However, the present study will be the first to investigate their integrated evolution as part of the natural history of PFP and its progression. In doing so, we will be able to determine their behavior in the long-term, as well as how they prospectively associate with each other and with clinical outcomes. Ultimately, this will provide a greater understanding of predictors of long-term outcome and possible targets for interventions.

## 1. Introduction

Patellofemoral pain (PFP) is characterized by an insidious onset of pain at the anterior/peri/retropatellar region of the knee (1). Its prevalence is approximately 25% in the general population and 35% in professional athletes (2). Disability, reduced physical activity, and impaired social life are reported in those with PFP (3, 4). Other impairments such as lower self-reported function, physical performance, and health-related quality of life (HRQOL) have also been reported in individuals with PFP, alongside with a possible progression to osteoarthritis (5–8).

PFP is multifactorial and involves a number of physical (e.g., kinetics, kinematics, muscle function) and non-physical features (e.g., psychological or lifestyle factors) (9–11). Proximal, local, and distal kinematics, kinetics, and muscle function impairments have been reported in those with PFP (1, 9, 12–17), and linked with increases in patellofemoral joint (PFJ) load during various tasks (9), a factor suggested to play a central role in PFP (9). Beyond this pathomechanical model of PFP (8), there is an increased awareness that non-physical features may be important in understanding PFP (18). Pain processing (e.g., local and central sensitization) and psychological features (e.g., kinesiophobia and pain catastrophizing) have also been reported to be altered in individuals with PFP (10, 11). Both physical and non-physical features are associated with worse pain, function, and disability (19–22). Some associations among physical and non-physical features with PFP are still not clear, including their prospective association in the long-term. The prospective association of PFJ load with PFP has also not been investigated, despite its proposed critical role (9).

PFP is not self-limiting and is challenging to manage, with symptoms persisting for nearly two decades. Long-term pain is reported to continue in one of every two patients even after treatment (3, 23). Symptom severity may remain unchanged or increase in 50% of affected individuals (24, 25). As such, a similar prospective pattern would also be expected for physical and non-physical features given their previously reported cross-sectional association with PFP. Symptoms duration of individuals with PFP has been previously associated with biomechanical (26) and muscle function parameters (27), as well as pain itself (27, 28). However, the majority of the studies conducted to date have a cross-sectional or interventional design, which precludes the understanding of the natural history of these features in individuals with PFP.

Understanding changes over time in physical and non-physical features in individuals with PFP is a first step to gain knowledge regarding their evolution as part of PFP and its progression. In addition, investigating the prospective association of clinical outcomes (i.e., self-reported pain, physical performance measures, self-reported function, HRQOL and physical activity level) with physical and non-physical features may contribute to the identification of predictors of pain and disability in the long-term, as well as possible targets for interventions.

We have, therefore, designed a prospective longitudinal study aiming to investigate group-by-time changes over a minimum of 15 months in individuals with PFP, measuring their biomechanical, muscle function, pain processing, psychological features and clinical outcomes. In addition, we aim to investigate the extent to which changes in biomechanical, muscle function, pain processing, and psychological features are associated with changes in clinical outcomes. We hypothesize that: (1) individuals with PFP will present worse biomechanical, muscle function, pain processing, psychological features, and clinical outcomes at follow-up as compared to baseline; (2) potential differences in outcomes as compared to asymptomatic individuals at baseline will increase at follow-up; (3) changes over the time in biomechanical, muscle function, pain processing, and psychological features will be associated with changes in clinical outcomes; (4) there will be an association between changes over the time in biomechanical and psychological features.

## 2. Methods

This study was approved by the local ethics committee of Sao Paulo State University (approval number: 4.6549.629). All participants provided written informed consent. Baseline assessments have already been performed, and participants are currently being followed up.

### 2.1. Participants

Participants aged 18 to 40 years were recruited through advertisements at social media, universities, gyms and public parks and divided in two groups: individuals with PFP (*n* = 144) and control individuals without PFP (*n* = 85). The following inclusion criteria were considered for PFP group (29): (i) Insidious symptoms of PFP lasting at least three months, (ii) worst knee pain in the last month of at least 20 mm on a 0–100 mm Visual Analogue Pain Scale (VAS), (iii) symptoms of PFP during activities that load patellofemoral joint (e.g., squatting, running, jumping, prolonged sitting, stair negotiation). To be included in the control group, participants had no signs or symptoms of PFP or other neurological or lower limb musculoskeletal conditions. Exclusion criteria for both groups were history of: patellar subluxation, surgery in any lower limb joint, trauma or injury at the knee, or ligament instability. Additional exclusion criteria for the follow-up assessments are: (i) occurrence of knee trauma or injury (both groups); (ii) development of knee pain (only for control group); (iii) development of other medical conditions that may influence the findings (both groups). Participants’ baseline demographics are presented in Table 1.

**Table 1:** Participants’ demographics at baseline.

Variables	PFP group (*n* = 144) Mean ± SD	Control group (*n* = 85) Mean ± SD
Females (*n*)	108	64
Males (*n*)	36	21
Age (years)	23 ± 5	22 ± 3
Height (cm)	166 ± 8	165 ± 9
Body mass (kg)	68 ± 14	62 ± 13
BMI (kg/cm^2^)	25 ± 4	23 ± 4

Abbreviations: SD, standard deviation; PFP, patellofemoral pain; BMI, body mass index.

### 2.2. Procedures

**Figure 1:**
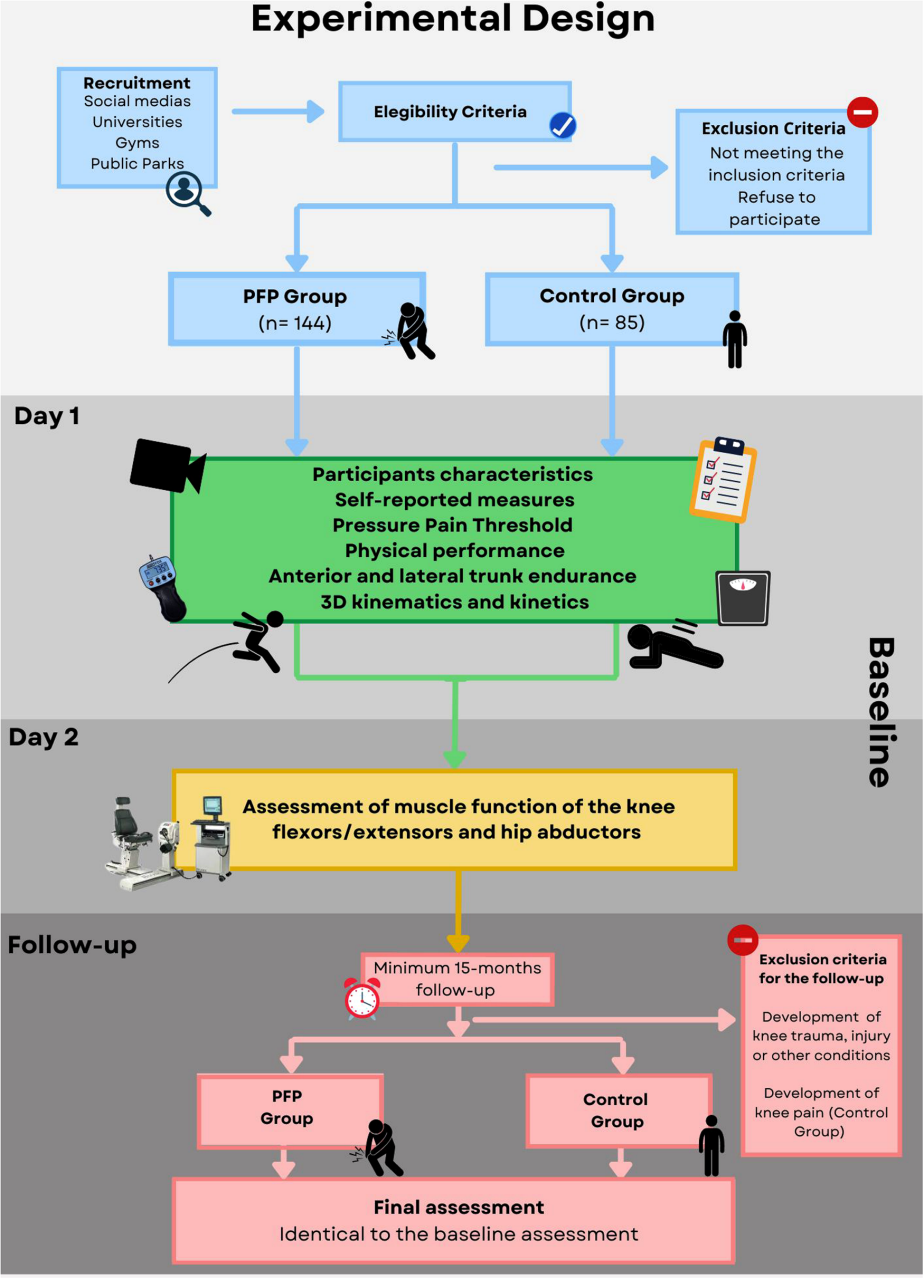
Flowchart depicting the experimental design of the study.

Follow-up assessments will be performed in two separate days, identically to the baseline (Figure 1). In the first day, characteristics of the participants, self-reported measures, Pressure Pain Thresholds (PPTs), physical performance measures, anterior and lateral trunk endurance, 3D kinematics and kinetics will be assessed. Participants will be oriented to perform all tasks barefoot and wear light clothes. In the second day, maximal torque, rate of torque development (RTD), and force steadiness of the hip abductors and knee extensors/flexors will be assessed. Physical performance measures and lateral trunk endurance will be assessed bilaterally, while PPTs, 3D kinematics and kinetics, and muscle function measures will be assessed in the symptomatic or most symptomatic knee (bilateral symptoms) in individuals with PFP. For controls, the assessed limb will be randomly selected. The same limb assessed in the baseline will be collected in the follow-up. At least 15 months after the baseline assessment, participants will be contacted by phone or e-mail and invited to return to the follow-up assessment.

### 2.3. Characteristics of the participants and self-reported measures

Age, pain laterality, and duration of PFP symptoms in months will be initially obtained. Weight and height will be assessed using a calibrated scale with a stadiometer (WELMY 110; WELMY, Brazil). Knee and ankle width will be measured using a pachymeter (150 mm × 0.05 mm/6”X1/128”; MARBERG, China) and lower limb length will be measured with a measuring tape from the anterior superior iliac spine to the medial malleolus. The anthropometric measures will be posteriorly included in the biomechanical model. Percentages of body fat and skeletal muscle mass will be assessed using a bioelectrical impedance analyzer (OMRON HBF 514C; OMRON Healthcare; Japan) and calculated with valid and reliable equations provided by the manufacturer (30). Subscapular, triceps, biceps and suprailiac skinfolds will be measured with a caliper (CESCORF TOP TEC; CESCORF, Brazil) following the recommendations of the International Standards for Anthropometric Assessment (31). The measures will be obtained along the right side of the body for 3 consecutive times, and the mean will be used for statistical analysis. Afterwards, participants will answer self-administered scales and questionnaires translated and cross-culturally adapted for the Brazilian population, which are detailed below (32–36).

#### 2.3.1. Self-reported pain

The self-reported knee pain will be assessed with the 0–100 mm VAS, where 0 represents no pain and 100 the worst pain imaginable. The worst level of pain during the last month, and the current level of pain (at the beginning of data collection) will be obtained. Participants will be also asked about their level of pain after biomechanical assessment and muscle function assessment. The VAS has been previously validated for individuals with PFP and has good reliability (intraclass correlation coeficient [ICC] = .76; standard error of measurement [SEM] = 6 mm; minimal clinically important difference [MCID] = 20 mm) (37).

#### 2.3.2. Self-reported function

Self-reported function will be assessed using the Anterior Knee Pain Scale (AKPS). The AKPS is a 13-item questionnaire that evaluates subjective symptoms and functional limitations in patellofemoral disorders (e.g., atrophy, subluxation, pain, swelling, difficulty performing daily activities). It has been validated for individuals with PFP and has good reliability (ICC = .81; SEM = 3.1 points; MCID = 10 points) (37, 38). The maximum score for each item ranges from 5 to 10, and the sum of all items generates a score ranging from 0 (maximal disability) to 100 (no disability).

#### 2.3.3. Kinesiophobia

Kinesiophobia will be assessed using the TAMPA Scale for Kinesiophobia (TSK). This is a valid and reliable tool developed to measure fear of painful movement or (re)injury (ICC = .82; SEM = 3.16 points; [MCID] = 4 points) (33, 39). The questionnaire consists of 17 items with statements related to fear of movement or (re)injury and participants are instructed to respond how much they agree with each of them, with ratings ranging from “strongly agree” to “strongly disagree”. The score of each item ranges from 1 to 4, and the sum of all items generates a final score ranging from 17 to 68, with higher scores representing higher kinesiophobia.

#### 2.3.4. Pain catastrophizing

Pain catastrophizing will be assessed using the Pain Catastrophizing Scale (PCS). This is a valid and reliable tool that assesses the excessively negative orientation towards actual or perceived pain (ICC = .90; SEM = 2.79 points; minimal detectable change [MDC] = 7.73 points) (34, 40). The questionnaire consists of 13 items with statements related to catastrophizing and participants are instructed to respond to what degree they describe their thoughts or feelings at the time of pain, with ratings ranging from “not at all” to “all the time”. The score of each item ranges from 0 to 4, and the sum of all items generates a final score ranging from 0 to 52, with higher scores representing higher pain catastrophizing.

#### 2.3.5. Health-related quality of life

The Medical Outcome Short-Form 36 (SF-36) will be used to assess HRQOL. This tool has been previously validated, and has satisfactory intra-rater reliability (*r* = .44 to.84) (41, 42). The SF-36 is composed of general questions related to eight domains: physical functioning, role physical, bodily pain, general healthy, vitality, social functioning, role-emotional and mental health. The questions result in a score for each domain ranging from 0 to 100, with higher scores representing better HRQOL. The eight domains will be summarized in the mental and physical components that will be calculated as per Taft et al. (43).

#### 2.3.6. Physical activity level

Self-reported physical activity level will be assessed using the Baecke's Habitual Physical Activity Questionnaire. This is a valid and reliable questionnaire (ICC = .92). The questionnaire consists of 16 questions related to type, duration and intensity of physical activities performed by the participants in the last 12 months. The questions result in three scores that represents three domains: physical activity at work, leisure practices and occupation of free time, and locomotion which range from 1 to 5 (44). Higher scores indicate higher levels of physical activity.

### 2.4. Pressure pain thresholds

PPTs are the minimum pressure stimulus perceived as painful and have been used to evaluate pain processing alterations (45)*.* PPTs will be assessed using a portable digital pressure algometer (WAGNER FORCE TEN FDX, United States) with a tip of one square centimeter. All measures will be performed by a single assessor trained to exert a pressure of 0.50 kgf/s (46). Participants will be positioned lying supine on a padded table. The algometer tip will be placed perpendicular to the skin at two points: (i) the center of the patella (local hyperalgesia), and (ii) the lesser tubercle of the humerus of the contralateral shoulder (widespread hyperalgesia) (46) (Supplementary Figure S1). Participants will be asked to report when the pressure sensation becomes painful (46). PPTs will be assessed twice at each site with a 30 s interval between assessments, and the mean will be used for statistical analysis. This protocol has been reported to have good intra-rater reliability in individuals with PFP (ICC = .72 to.80; SEM = .41 to.55 kgf/s; MDC = 1.14 to 1.52 kgf/s) (47).

### 2.5. Physical performance

The Single Leg Hop Test and Forward Step-Down Test will be obtained as measures of physical function. Prior to testing, three familiarization trials will be performed to minimize learning effects. To perform the Single Leg Hop Test, participants will be positioned standing on the tested leg, with the non-tested knee flexed at 90° and the arms crossed behind their back. They will be asked to hop forward as far as possible, landing on the same leg while maintaining their balance (14, 15) (Supplementary Figure S2). The distance between the initial and final heel positions will be recorded in centimeters with a measuring tape. If participants lose their balance or swing their arms, the trial will not be considered valid and will be repeated (14, 15). The mean of three valid repetitions will be used for statistical analysis. Reliability of this test has been reported to be excellent (ICC = .96; SEM = 4.56 cm) (48). For the Forward Step-Down Test, participants will be positioned standing with their test leg on a step, their hands on their waist, and their non-test leg in front of the step with the knee extended and the ankle dorsiflexed. The step height will be standardized so that all participants achieve 60° of knee flexion during testing (49). Participants will be asked to tap the floor with their non-test heel and then return to the starting position (15) (Supplementary Figure S3). Participants will be asked to perform as many repetitions as possible in 30 seconds and the number of repetitions will be recorded (15). Repetitions where the participant does not tap the floor, loses their balance, or change the position of their hands will not be considered valid and will not be added to final test score (15). The Forward Step-Down Test will be performed once without interruptions. Reliability of this test has been reported to be excellent (ICC = .94; SEM = .53 repetition) (50).

### 2.6. Trunk muscle endurance

Anterior and lateral trunk endurance will be assessed with the Prone-Bridge and Side-Bridge tests, respectively. For the Prone-Bridge test, participants will be initially positioned lying prone on an exercise mat, propped on their forearms and feet, with shoulders and elbows flexed at 90°. Arms will be shoulder-width apart and feet will be hip-width apart (15). Participants will be asked to raise the pelvis from the floor, maintaining this static position as long as possible (15) (Supplementary Figure S4). For the Side-Bridge test, participants will be positioned side-lying on an exercise mat, with their hips in a neutral position, legs extended and both feet in tandem on the exercise mat. The foot on the side being tested will be positioned behind the contralateral foot, in a staggered position. The support arm will be placed vertically with the elbow aligned with the shoulder, and the contralateral hand placed on the waist (15). Participants will be asked to lift their hips off the mat, and to hold the static position as long as possible (15) (Supplementary Figure S5). Both tests will be stopped when participants no longer sustain the test position and the duration in seconds will be recorded (15). Verbal encouragement will be not provided during the test, although one verbal correction might be given to correct participants’ positioning if necessary. Reliability of these tests has been reported to be excellent (ICC = .91 to.96; SEM = 4.79 to 5.46 s; MDC = 13.28 to 15.13 s) (51).

### 2.7. Biomechanics

Kinematics and kinetics data during single leg squat, step-down and single leg hop tasks will be collected using a 5-camera motion analysis system at 100 Hz (VICON Motion Systems, United Kingdom) synchronized with a force plate at 4000 Hz (4060; BERTEC, United States). Retroreflective markers (14 mm) will be placed according to the Plug-in-Gait model, with one additional maker on the medial knee (Supplementary Figure S6). Upon marker placement, a standing calibration trial will be performed, and the knee joint center will be determined using a virtual Knee Alignment Device (KAD) to minimize *cross-talk* errors. Participants will be then instructed to perform three practice trials of each task for familiarization. For the single leg squat task, participants will be asked to stand with their tested leg on the force plate and the non-tested knee flexed at 90°. They will be instructed to squat to an angle greater than 60° of knee flexion, and then to return to the initial position (Supplementary Figure S7). The step-down task will be performed similarly to the Forward Step-Down Test except that it will be performed on the force plate and without instructions to perform as many repetitions as possible (Supplementary Figure S8). Five trials of the single leg squat and step-down tasks will be obtained. For the single leg hop task, participants will stand on the force plate, with their hands on the waist. Participants will be instructed to hop forward as far as possible with their test leg, landing on the same leg (propulsion phase, Supplementary Figure S9). The same procedure will be then performed with the participant landing on the force plate (landing phase, Supplementary Figure S10). Three trials of each phase will be obtained, six in total.

### 2.8. Muscle function

Maximal torque, RTD and force steadiness will be collected with an isokinetic dynamometer with an acquisition frequency of 100 Hz (System 4 Pro; Biodex, United States). The starting muscle group (hip abductors or knee extensors/flexors) and the type of contraction (isometric, concentric or eccentric) will be randomized.

For the assessment of the knee extensors/flexors, participants will be seated with their hips and non-tested knee flexed at 90°. The dynamometer axis will be aligned with the center of the knee joint. Two belts crossing the trunk, one around the pelvis, and another on the distal tested thigh will be used to stabilize the participants (13, 14) (Supplementary Figure S11). The isometric torque will be tested at 60° of knee flexion, while concentric and eccentric torques will be tested from 20° to 90° of knee flexion with an angular velocity of 30°/seconds (13, 14). For the hip abductors assessment, the participants will be positioned in a side-lying position with the test leg on top of the non-test leg and, hips in neutral sagittal and transverse plane positions. The dynamometer axis will be aligned to the anterior superior iliac spine at the level of the greater trochanter (13). Four belts will be used to stabilize non-test leg and the trunk (Supplementary Figure S12). The isometric torque will be tested at 30° of hip abduction and concentric and eccentric torques will be tested from 0° to 30° of hip abduction with an angular velocity of 30°/seconds (13).

Maximal torque and RTD will be assessed as follows. For the isometric contraction familiarization, two submaximal contractions of six seconds will be performed, with one-minute interval between them (13, 14). Afterwards, two maximal contractions of six seconds will be collected, with a three-minute interval between them (13, 14). For concentric and eccentric contraction familiarization, five submaximal contractions and two maximal contractions will be performed with one-minute interval between them (13, 14). Then, three maximal contractions of six seconds will be collected, with a three-minute interval among them (13, 14). Participants will be verbally encouraged to perform maximal contractions and visual feedback exhibiting torque-time curve will be provided (13).

Force steadiness will be assessed during sub-maximal isometric force-matching tasks. The target torque will correspond to 10% of maximal torque estimated from the isometric test (12, 52). The dynamometer monitor will display the targeted torque simultaneously with the torque generated by the participant. Participants will be instructed to match their generated torque with the target torque and keep it as steady as possible. Two familiarization trials of 10 seconds will be performed, with a 30 s interval between them (12, 52). Then, three trials of 20 seconds, with one-minute interval among them will be collected (12, 52).

### 2.9. Data analysis

**Figure 2:**
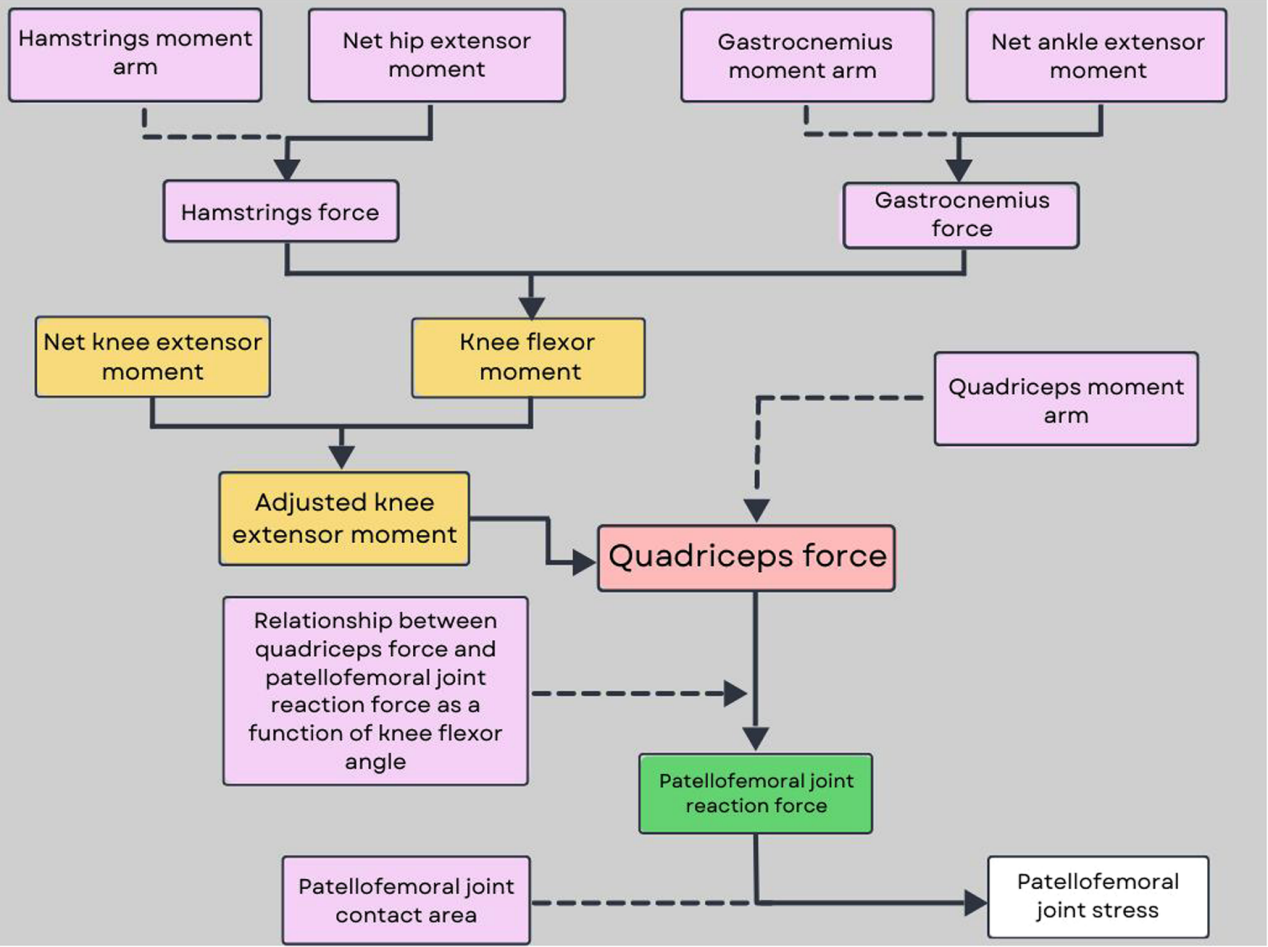
Flowchart of the PFJ model.

A Woltring filter with a two mean square error will be applied to reduce the vibratory noise that could arise during the marker trajectories due to soft tissue artefact marker trajectory in Vicon Nexus (2.12; VICON Motion Systems, United Kingdom). Vertical ground reaction force (VGRF) will be filtered at 10 Hz using a zero-lag fourth-order low-pass filter. For single leg squat and step-down tasks, the beginning of the task will be defined as the moment when the participant starts performing the descent phase of the task (measured *via* the increase in knee flexion of the tested limb), while the end of the task will be defined as the moment when the participant returned to double leg stance (52). For single leg hop task, landing phase will be defined from initial contact (instant when VGRF first exceeds 10N) to peak knee flexion, while propulsion phase will be defined from peak knee flexion to take off (instant when VGRF first decreases below 10N). Hip, knee, and ankle joint kinematics will be calculated using a joint coordinate system approach, while trunk and pelvis kinematics will be calculated relative to the laboratory coordinate system. Peak and range of motion of trunk, pelvis, hip, knee and ankle kinematics in the sagittal and frontal planes will be calculated. Net joint moments will be also computed using standard Newton-Euler inverse-dynamics equations and will be expressed as internal moments normalized to each participant’s body mass (53). PFJ contact force and stress will be also calculated based on a previously described biomechanical model proposed by Devita and Hortobagyi (54). In this model, hip, knee and ankle joint angles and moments are used to calculate hamstrings, quadriceps and gastrocnemius muscle force. In doing so, the knee extensor moment is adjusted by the co-contraction of the knee flexors (55) (Figure 2). PFJ stress will be then calculated for the entire task, but the peak value will be the variable of interest.

Maximal isometric, concentric and eccentric torques will be determined as the highest value obtained during each contraction type and will be normalized by the body mass of each participant ([N·m·kg^−1^] × 100) (17, 52). RTD will be calculated by dividing normalized torque variation by the time variation (ms) from the onset of the contraction up to 30% and 60% of maximal torques (17, 52). Contraction onset will be defined as the point at which the torque curve exceeded the baseline value by 2% of maximal torque (17, 52). Force steadiness will be determined by the coefficient of variation of torque for each trial (torque standard deviation/mean torque × 100) and the mean of the 3 trials will be used for statistical analysis (12, 52). The first and last 3 seconds of contraction will not be considered for analysis (12, 52). A custom-written code in MATLAB will be used to obtain all variables of interest (R2017a; The MathWorks, Inc, United States).

### 2.10. Statistical analysis

Descriptive statistics (mean, median, standard deviation, and interquartile intervals) will be calculated for all variables of interest. Generalized Linear Mixed Model (GLMM) will be used to investigate group-by-time differences. The type of matrix will be chosen according to the Akaike Information Criterion (AIC) in order to assess the adherence index of the model (56). The lower the AIC, the higher the adherence (56). Group and time interaction effects will be considered fixed, while participant effects will be considered random. Pairwise comparisons will be performed with Bonferroni *post hoc* tests when significant interactions or main effects are determined. Mean differences [95% confidence intervals (CI)] and effect sizes (Cohen’s *d* [95% CI]) for each *post hoc* comparison will be calculated. The guidelines for interpreting the Cohen’s *d* are: 0 to 0.40 small effect, 0.41 to 0.70 moderate effect, 0.71 or large effect (57). Statistical significance level will be set at .05.

For the first stage of the regression analyses, univariate linear regression models will be used to determine to what extent changes in each biomechanical, muscle function, pain processing and psychological features are associated with changes in self-reported pain, physical performance measures, self-reported function, HRQOL and physical activity level in individuals with PFP. A significance level of *p* ≤ .10 rather than the conventional level of *p* < .05 will be used to ensure that the univariate analyses are sufficiently sensitive to identify potential prognostic factors for entry in the model (58). For the second stage, all potential prognostic factors that showed significant associations on univariate analyses will be entered into a stepwise multivariate linear regression with backward elimination (*p* ≤ .10) in order to identify a group of factors that are independently associated with the clinical outcomes. For the final multivariate models, the significance will be set at .01 to minimize the results being adversely influenced by the likelihood of increased risk of Type I error associated with multiple analyses. The predictive power of each final model will be determined by calculation of the percentage of explained variance (adjusted *R*^2^).

## 3. Discussion

This is a protocol of an ongoing prospective longitudinal study designed to investigate group-by-time changes over a minimum of 15 months follow-up in PFP symptoms, biomechanical, muscle function, pain processing, and psychological features; and to investigate the extent to which changes in biomechanical, muscle function, pain processing, and psychological features are associated with changes in self-reported pain, physical performance measures, self-reported function, HRQOL, and physical activity level.

Several cross-sectional studies have reported impairments in physical and non-physical features in individuals with PFP (9). The impairments reported so far include: local, proximal and distal joint kinematics alterations such as lower knee flexion, greater hip adduction, and rearfoot eversion (59); joint kinetics alterations such as lower knee extensor moments and higher knee abduction moments (21, 60); lower strength, power and steadiness of the hip abductors, knee extensors and flexors (12, 13); high levels of kinesiophobia and pain catastrophizing (61); local and widespread hyperalgesia (11). However, these features were generally investigated as independent factors and their interaction remains to be further investigated.

The pathomechanical model of PFP proposed that altered joint kinematics and kinetics may be driven by impairments in muscle function, which would ultimately lead to increased PFJ load and pain (9). However, recent studies have proposed that non-physical features may also play a role in the altered joint biomechanics, load, and symptoms (62). For instance, fear avoidance beliefs have been associated with single leg squat hip adduction, step-down knee abduction, jogging knee abduction, and jogging hip adduction in women with PFP (63). Associations between PPTs and step-down knee abduction have also been reported in individuals with PFP (64). Although the findings from these studies contribute to the understanding of the interaction between physical and non-physical features in individuals with PFP, there is still a gap in the knowledge regarding how this interaction progresses prospectively during the natural course of PFP.

The investigation of the natural course of PFP can provide additional insights. For instance, PFJ stress has been suggested to play a central role in PFP pathophysiology (9). However, its behavior over time in individuals with PFP has never been investigated. As physical and non-physical features are thought to contribute to PFJ stress (9, 63–65), our study will be able to provide data regarding which features have associations with changes in PFJ stress in the long-term.

This study has some limitations that should be acknowledged. Only young adults were included in our study, limiting the generalizability of our findings to adolescents and older individuals. Our study will provide insights regarding the natural history of physical and non-physical features in individuals with PFP, however, cause and effect cannot be established given the study design. Lastly, this prospective study includes assessments in only two time points (i.e., baseline and follow-up). As PFP is characterized by intermittent symptoms (1), this may have an influence on the results.

## Data Availability

The original contributions presented in the study are included in the article/**Supplementary Material**, further inquiries can be directed to the corresponding author/s.
