# Supplementary material for: Patellofemoral pain over time: Protocol for a prospective, longitudinal study investigating physical and non-physical features
Source: Front Sports Act Living. 2023 Jan 11;4:1081943. doi: 10.3389/fspor.2022.1081943 (PMC9875135; doi:10.3389/fspor.2022.1081943)
Supplement: Supplementary file 1 [file Datasheet1.pdf]

Supplementary Material

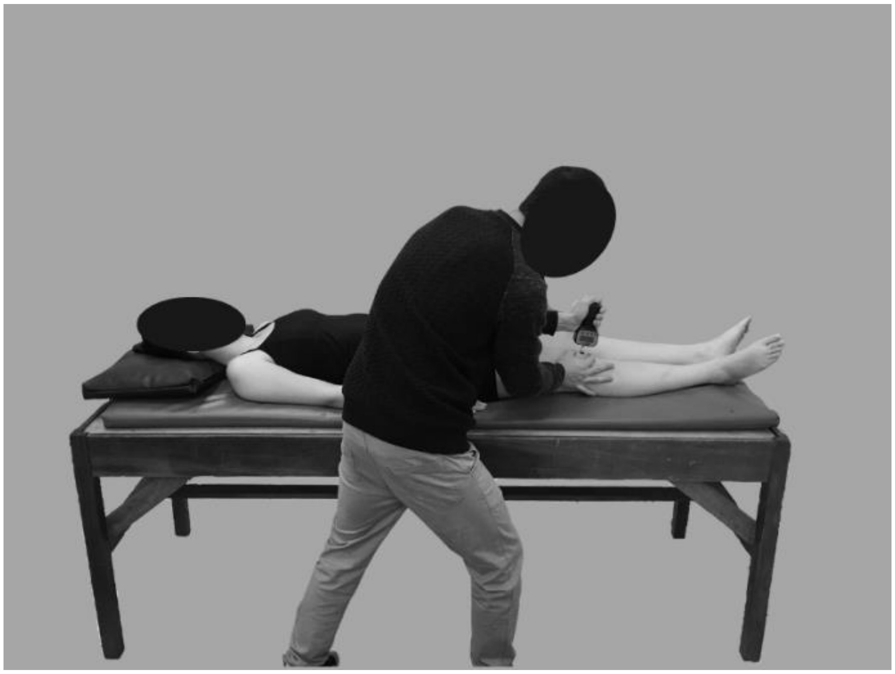

Figure S1a: Pressure Pain Thresholds in the center of patella.

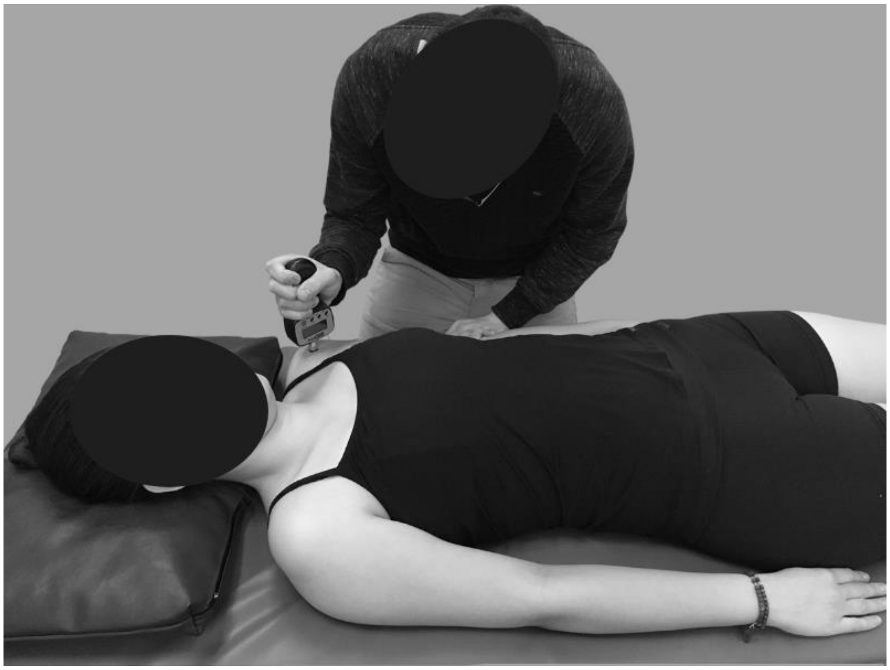

Figure S1b: Pressure Pain Thresholds in the contralateral shoulder.

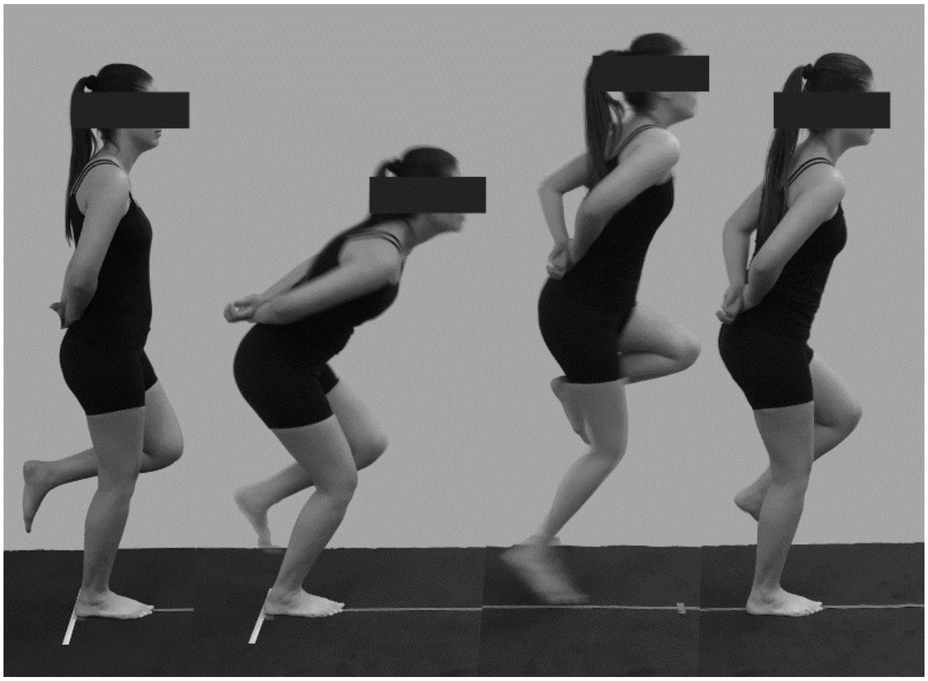

Figure S2: Single-Leg Hop Test.

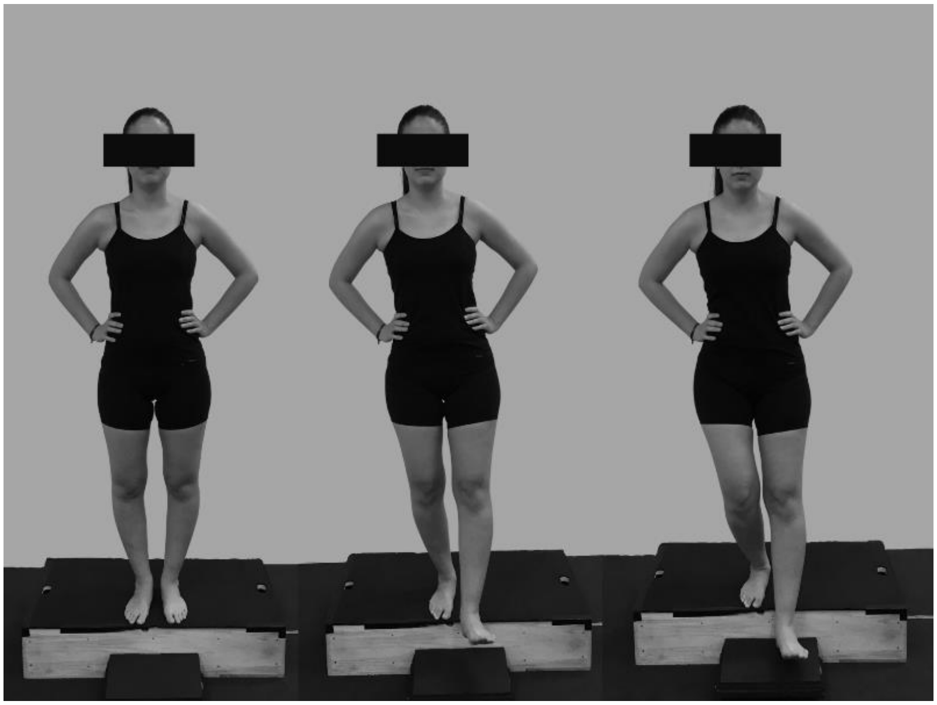

Figure S3: Forward Step-Down Test.

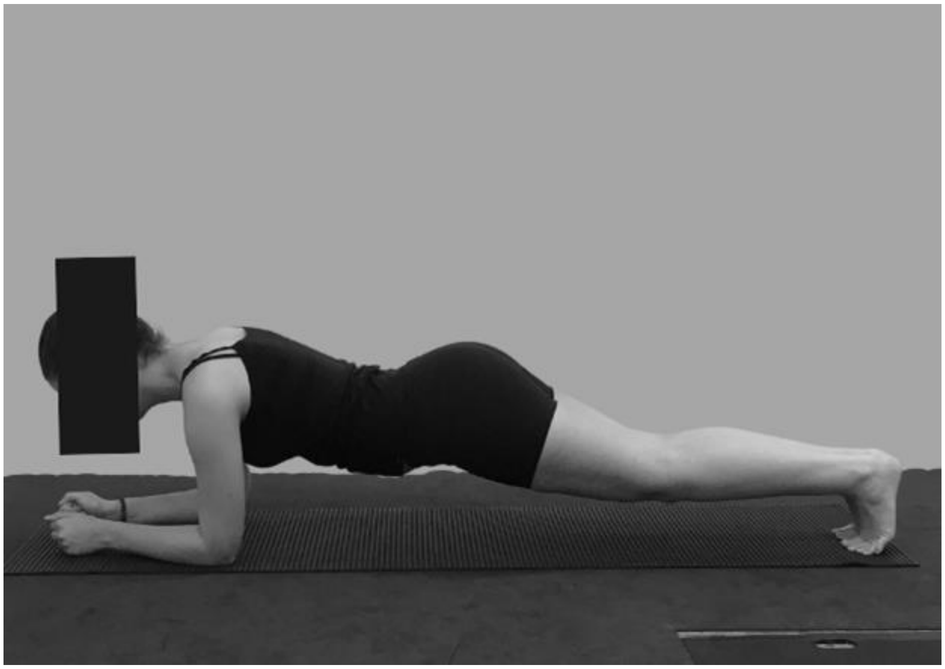

Figure S4: Prone-Bridge test.

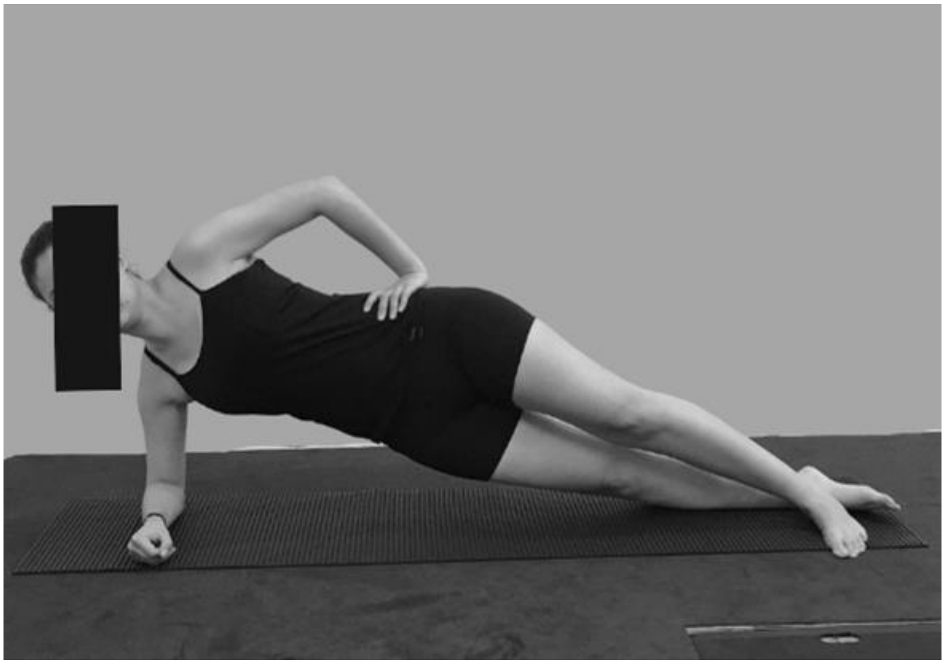

Figure S5: Side-Bridge test.

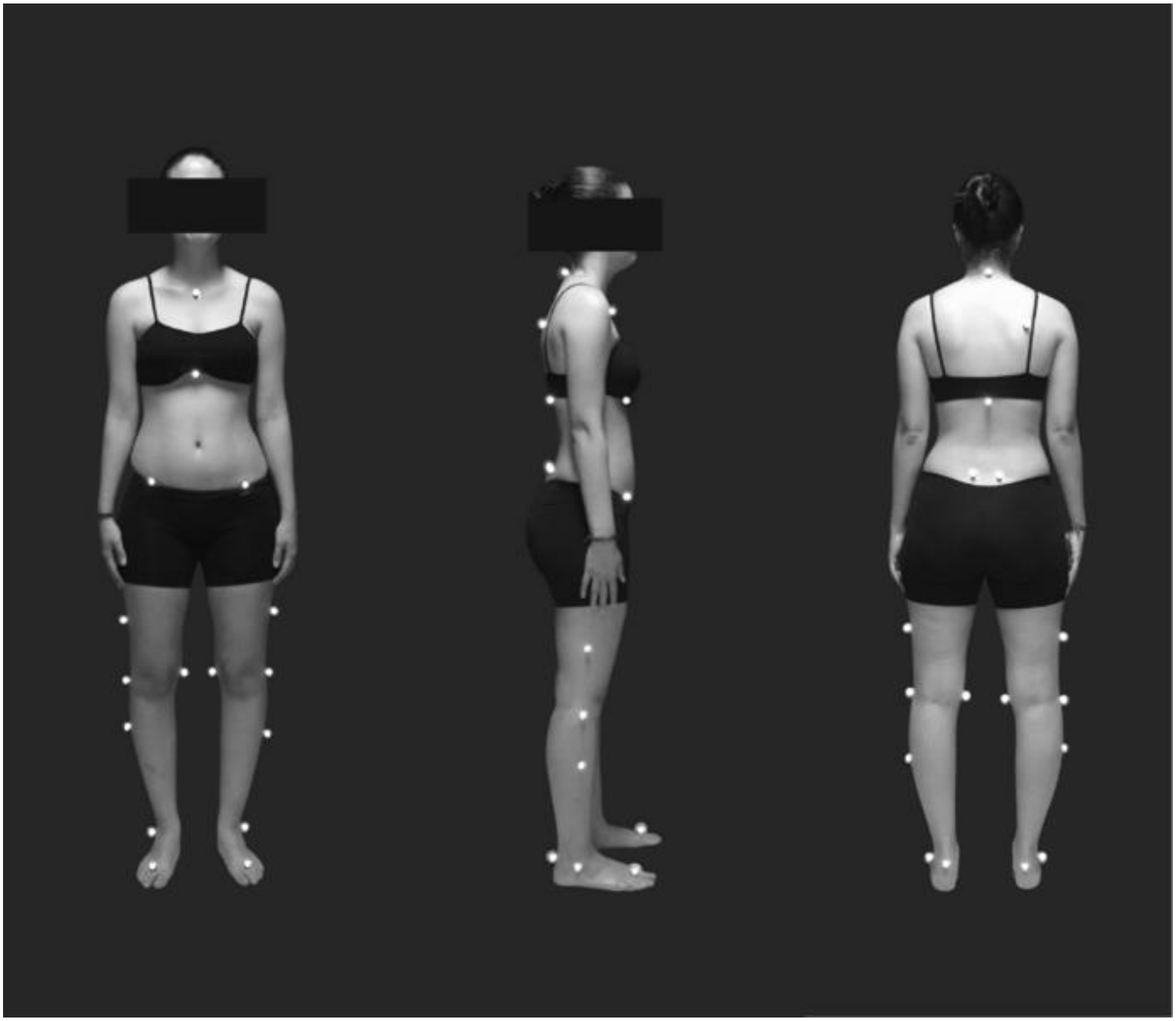

Figure S6: Biomechanical model.

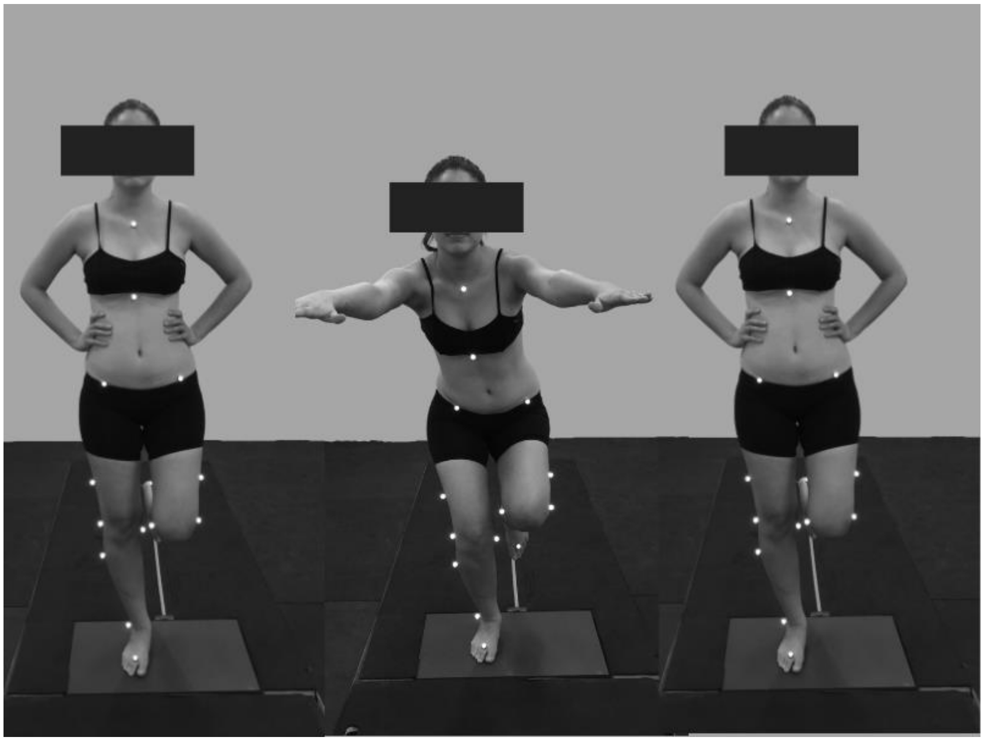

Figure S7: Single Leg Squat.

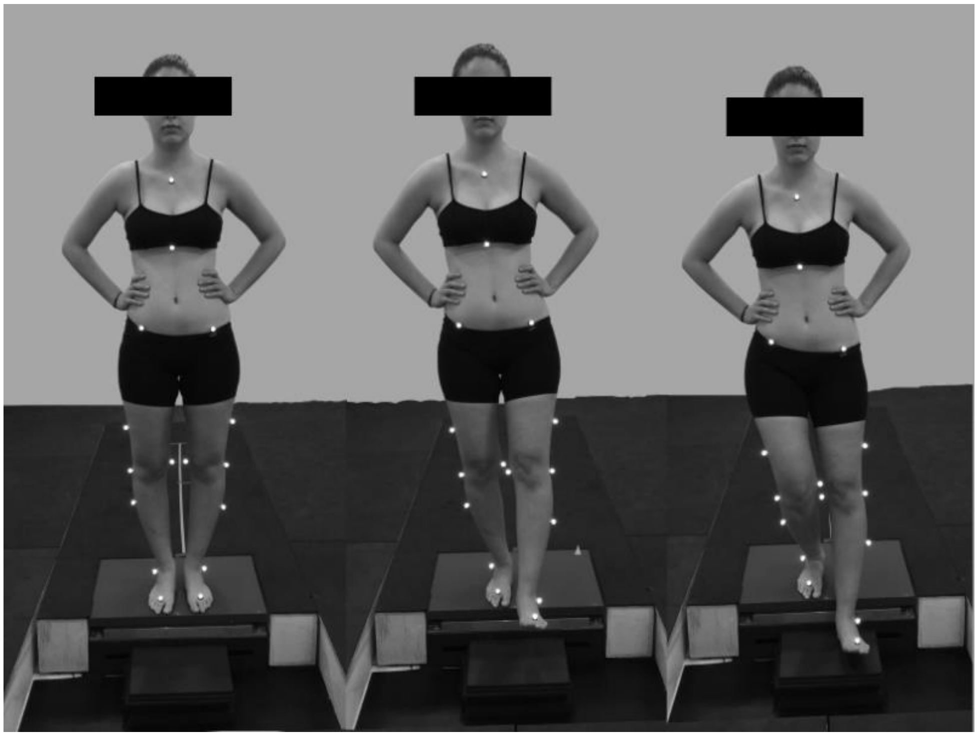

Figure S8: Step-Down.

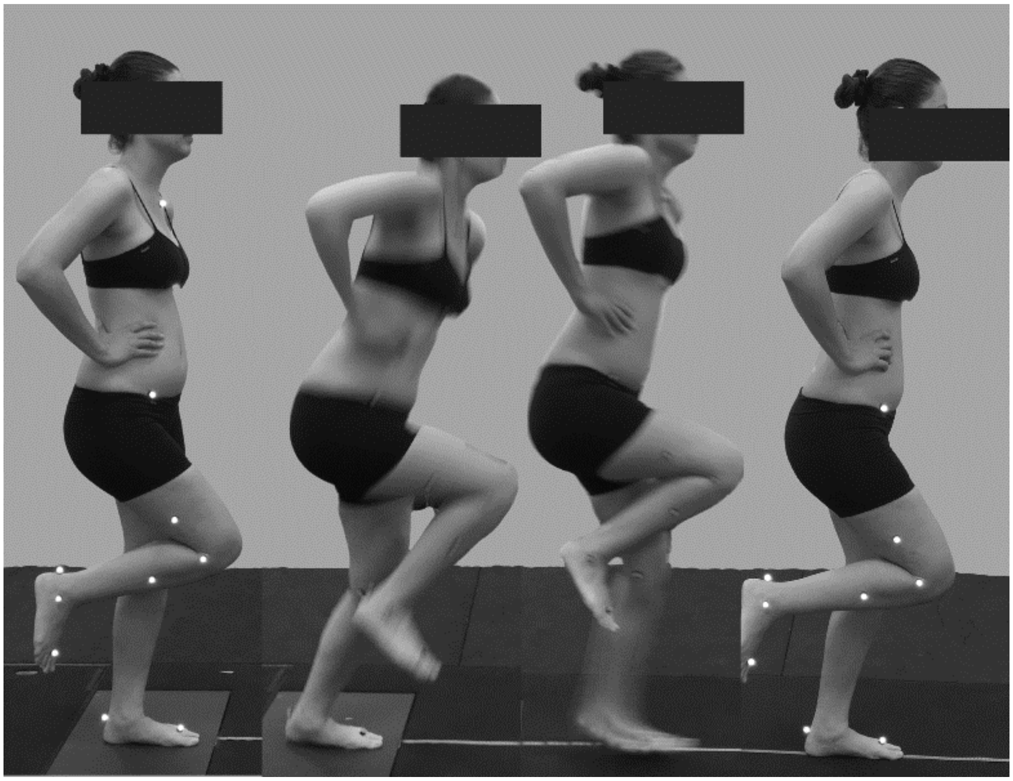

Figure S9: Single Leg Hop (propulsion phase).

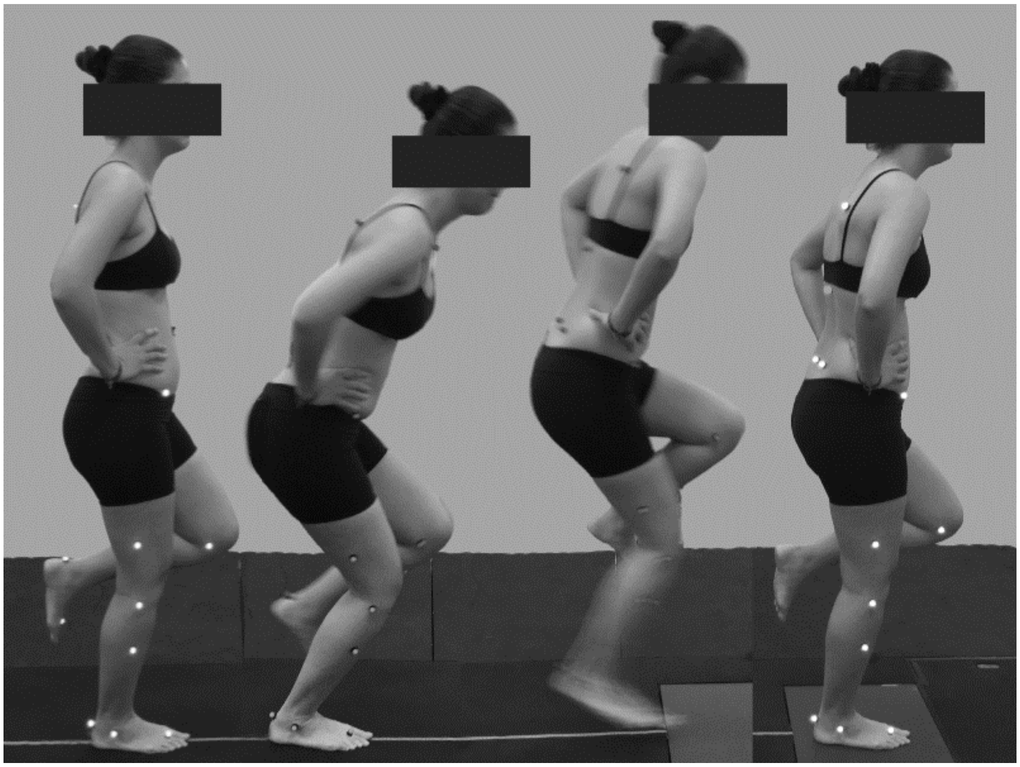

Figure S10: Single Leg Hop (landing phase).

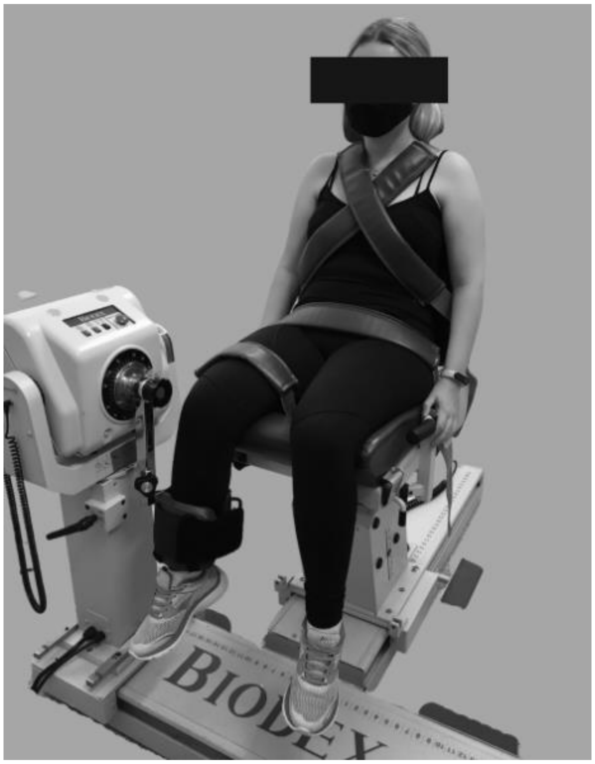

Figure S11: Position for the strength measures assessment of the knee extensors/flexors.

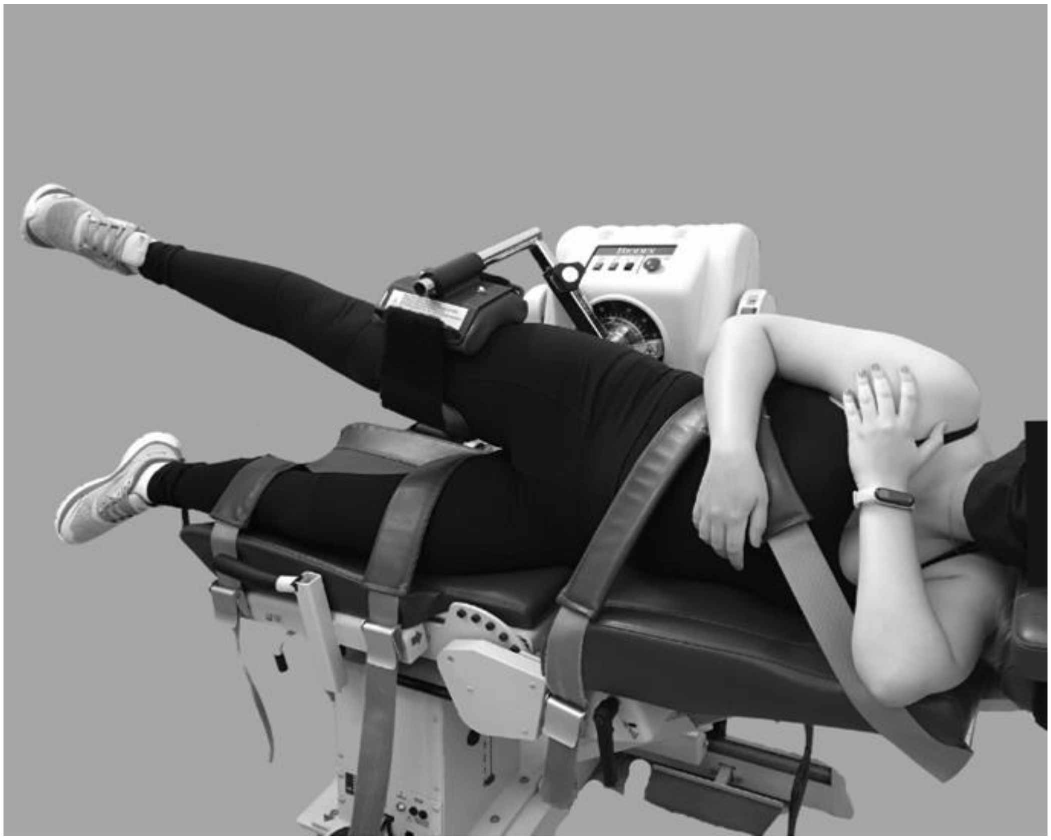

Figure S12: Position for the strength measures assessment of the hip abductors.
